# Fire Retardant Phase Change Materials—Recent Developments and Future Perspectives

**DOI:** 10.3390/ma16124391

**Published:** 2023-06-14

**Authors:** Kinga Pielichowska, Natalia Paprota, Krzysztof Pielichowski

**Affiliations:** 1 Department of Biomaterials and Composites, Faculty of Materials Science and Ceramics, AGH University of Science and Technology, Al. Mickiewicza 30, 30-059 Kraków, Poland; npaprota@agh.edu.pl; 2 Department of Chemistry and Technology of Polymers, Faculty of Chemical Engineering and Technology, Cracow University of Technology, ul. Warszawska 24, 31-155 Kraków, Poland; krzysztof.pielichowski@pk.edu.pl

**Keywords:** phase change materials, flammability, paraffins, fatty acids, poly(ethylene glycol), battery thermal management

## Abstract

The accumulation of thermal energy in the form of latent heat of phase transition using phase change materials (PCMs) is one of the most attractive and studied research areas with huge application potential in both passive and active technical systems. The largest and most important group of PCMs for low-temperature applications are organic PCMs, mainly paraffins, fatty acids, fatty alcohols, and polymers. One of the major disadvantages of organic PCMs is their flammability. In many applications such as building, battery thermal management, and protective insulations, the crucial task is to reduce the fire risk of flammable PCMs. In the last decade, numerous research works have been performed to reduce the flammability of organic PCMs, without losing their thermal performance. In this review, the main groups of flame retardants, PCMs flame retardation methods as well as examples of flame-retarded PCMs and their application areas were described.

## 1. Introduction

**Figure 1:**
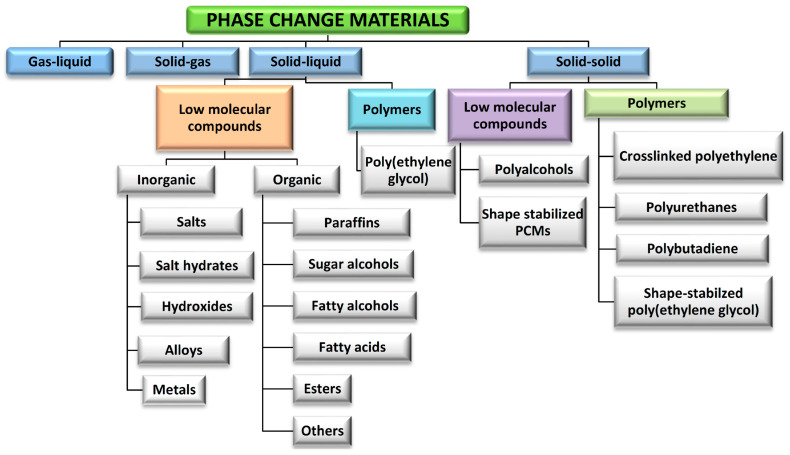
Classification of PCMs. Adapted with permission from Ref. [4]. 2014, Elsevier.

The growing demands on energy and the limited amount of resources have accelerated research efforts in the field of energy production, especially from renewable resources, and its storage. Importantly, a considerable amount of energy generated from renewable resources and waste heat from district heating and industry cannot be used due to the lack of efficient and advanced technologies and systems that allow efficient heat storage or conversion [1]. With properly designed thermal energy storage (TES) systems, heat energy can be collected, stored, and released according to the factual requirements and demands. TES systems can store thermal energy as sensible heat energy storage, latent heat energy storage, and chemical heat energy storage. The accumulation of thermal energy in the form of latent heat of the phase transition is one of the most attractive and extensively studied research areas [2,3]. In such systems, heat is stored in phase-change materials that can absorb and release large amounts of energy in the form of latent heat during phase change. Phase change materials (PCM) are one of the most important groups of materials that have been used for the storage of thermal energy [4,5,6]. PCM-based systems are characterized by a high energy storage density and keep a constant temperature in the heat storage process during the phase transition. PCMs can be classified by the type of phase transition as gas–liquid, solid–gas, solid–liquid, and solid–solid systems. However, due to the large volume difference between solid or liquid phase and gaseous phase, PCM with solid-liquid and solid-sold phase transition have been studied and applied in the practice. Over the last few decades, different groups of PCMs have been studied, modified, and tested—Figure 1.

PCMs absorb and release large amounts of thermal energy during the phase transition process in the form of latent heat as well as in the form of sensible heat before and after the phase transition. From a historical point of view, the first generation of PCMs were salts and salt hydrates. PCMs based on salts have a high latent heat and are cost-effective, but they promote corrosion in metallic environments in practical applications. The second generation of PCMs were organic PCMs, especially paraffins and fatty acids. They are characterized by good stability, high storage capacity, and low vapor pressure in the melt; however, their thermal conductivity is low and they are flammable. The third generation of PCMs includes polymeric PCMs, as well as metals with high electrical conductivity and optical properties [2]. On the other hand, the application of organic and polymeric PCMs can provide benefits to thermal storage in many technical applications [7]. However, the serious problem is their flammability that limits widespread use in many growing sectors, such as energy efficient buildings and electric vehicles.

Hence, in this review, main routes to reduce the flammability of organic PCMs, examples of flame-retarded PCMs, and their application areas will be discussed.

## 2. Flame Retardants

Combustible volatiles are generated by the majority of organic compounds and polymers in the presence of heat and oxygen. In order to diminish fire risk and improve fire safety, different strategies have been developed to prevent and limit flammability and lower the amount of heat released during the burning of organic and polymeric materials. One of the most popular ways is the application of flame retardants (FR), including intumescent FRs (IFRs) [8]. The different types of flame retardants are presented in Table 1.

**Figure 2:**
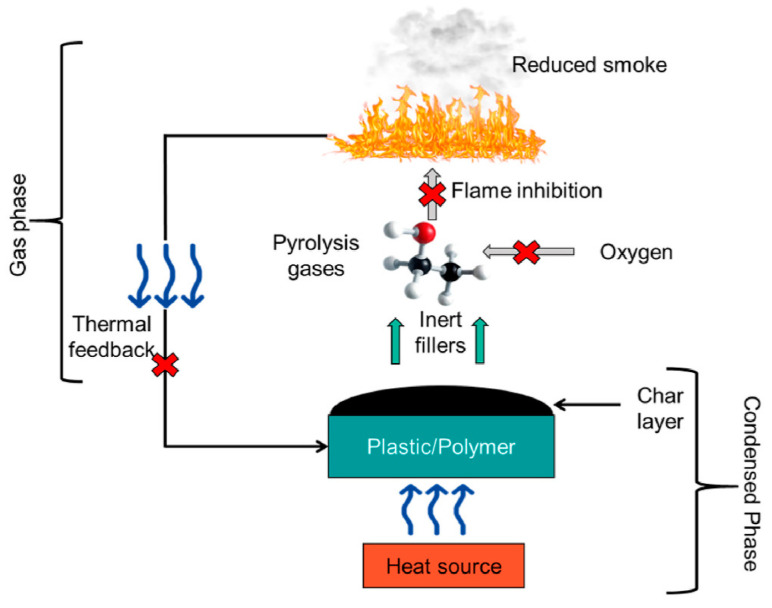
Schematic diagram that illustrates the flame retardant mechanisms. Reprinted/adapted with permission from Ref. [10]. 2022, Elsevier.

The mechanism and mode of action of FR in polymeric materials depend on their incorporation methods and the types of chemicals used as FR. In general, flame retardancy mechanisms can be classified as those operating in gaseous, condensed, and in both phases—Figure 2.

In the gas phase, flame retardant can inhibit the oxidation of hydrocarbons derived from the pyrolysis of materials and reduce the heat released during combustion. Incorporation of flame retardants can also lead to the release of inert gases at high temperature that dilute the combustible mixture and delay ignition. Other modes utilize the effect of the catalyst inhibiting flaming combustion or the introduction of FR which can act as a heat absorber and reduce the temperature of the materials to a safe level.

In the condensed phase, upon heat exposure, the material with incorporated FR undergoes thermal degradation and dehydration. These processes result in cross-linking and the formation of a carbon char layer on the surface of the material. The burning rate of the material decreases as the char layer acts as an insulating shield [10]. The main groups of flame retardants are intumescent flame retardants, heat absorbers, flame quenchers, and synergist systems.

Intumescent flame retardants (IFRs) work in the condensed phase and form a foam and char layer on the surface of the material and prevent the underlying material from further exposure to ignition [9] and the release of combustible compounds. Usually, IFR system consists of three components: acid source (e.g., melamine polyphosphate (MPP), ammonium polyphosphate (APP)), carbonization agent (hydroxyl-containing compounds, e.g., polyols, pentaerythritol (PER)), and blowing agent that release gas under heating (e.g., melamine and urea) [11].

IFRs are considered to be more environmentally friendly alternatives to halogenated hydrocarbons because they release fewer toxic gases during combustion.

The next group of FRs are heat absorbers. Among them, especially magnesium or aluminum hydroxide, magnesium carbonate, or mixed magnesium/calcium carbonates and hydroxides are considered sustainable and environmentally friendly fire retardants [12]. Heat absorbers undergo endothermic decomposition with release of low-molecular weight volatiles, such as water, ammonia, and carbon dioxide. They dilute the fuel supplied to the combustion zone, and there is a decrease in the risk of fire. In addition, depending on the FR composition, they can also form a protective layer of the corresponding oxides. A disadvantage of such materials is the relatively high loading (up to 70%) that is often required to obtain proper effectiveness—such a high load can deteriorate the mechanical properties of the polymer composite [9].

**Scheme 1:**
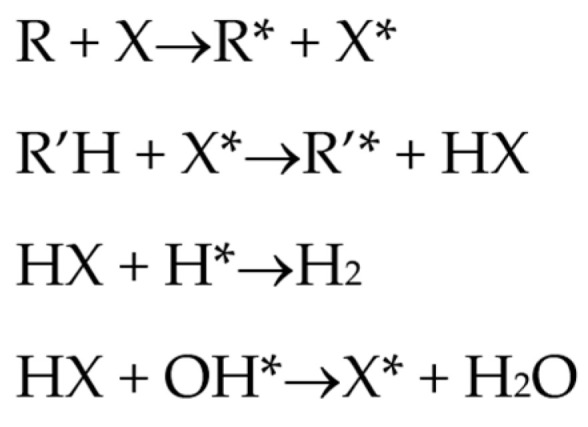
Reactions of HFR with OH and H radicals under heat exposure [13].

Halogenated flame retardants (HFRs) are one of the most important and largest FR groups; however, they raise serious environmental concerns. Among all HFRs, compounds with bromine are considered very effective in flame retardancy. Under heat exposure, they interrupt polymer combustion in the gas phase by producing free radicals in a continuous way [13]. Free radicals released from halogenated FRs (RX) react with OH and H radicals that are generated during the combustion of organic materials as presented in Scheme 1.

The key factor influencing the effectiveness of HFR is the type of halogen. Usually, FRs with bromine and chlorine atoms are used, while fluorine compounds are less common since fluorine radicals are usually released at quite high temperatures, much higher than the decomposition temperature of most organic materials [13]. A few examples of HFRs are presented in Table 2.

Another group of FRs are synergist systems. In such systems, compounds with minimal intrinsic fire retardancy, but the ability to considerably improve the fire retardancy of other compounds, are applied. In practice, combinations of two or more fire retardants used together exert a stronger effect than the sum of individual components [9]—Table 1. Synergistic FR may include nanoparticles to achieve a good flame retardancy effect; e.g., Didane et al. [14] investigated the fire behavior of PET modified with zinc phosphinate and nanostructured polyhedral oligomeric silsesquioxanes (POSS). Isitman and Kaynak [15] reported on the synergistic flame retardance effect in poly(methyl methacrylate) after application of organically modified layered-silicate nanoclays and multiwalled carbon nanotubes together with an organophosphorus FR.

## 3. PCMs Flame Retardation Methods

Flame retardants can be applied to PCMs using different approaches, such as

- incorporation into bulk PCM-FR is directly incorporated and mixed with PCM; this strategy allows for reducing the flammability of bulk PCMs such as paraffin and fatty acids; however, phase separation can occur and material still needs to be shape stabilized or encapsulated;
- insertion of FRs into the PCM matrix—FR is incorporated into the polymer matrix that is applied for PCM shape stabilization (e.g., high-density polyethylene, epoxy resin, polyurethane foam);
- FR is incorporated into the polymer shells and PCM is closed inside the capsules, or FR is located in the microcapsules and then dispersed in bulk PCM;
- flame retardancy of shape-stabilized phase change materials (SSPCMs) by surface coating—shape-stabilized PCMs are surface coated by the flame retardant system.

## 4. Flame-Retarded PCMs

Depending on the type of PCM, different FR and strategies need to be applied to achieve the flame retardation effect. In the next subsections, flame retardancy methods for different PCMs will be described. Selected examples of flame-retarded PCMs are presented in Table 3.

Total heat release (total HR), peak heat release rate (peak HRR), and limiting oxygen index (LOI) are parameters used to characterize the flammability of the material. Total HR is the amount of heat energy released by the material during its burning process; usually expressed as surface area normalized energy (J/m^2^). The peak HRR defines the maximum release rate of heat during the combustion of the sample. The peak HRR occurs when the decomposition process takes place at its highest rate and is considered a dependable measure of maximum flammability. The total HR and peak HRR are determined using the cone calorimeter [16]. The LOI parameter indicates the minimum oxygen concentration (in vol%) that is indispensable to support the flaming combustion of the material after ignition. The lower the LOI parameter, the more flammable the material. It is measured by passing a mixture of oxygen and nitrogen over a burning sample and reducing the oxygen level until a critical (minimal) level is reached [17].

**Table 3:** Flame-retarded PCMs.

PCM	Flame Retardants	Latent Heat J/g (MP °C)	Mass Residue % (Temp.)	Total HR (MJ/m^2^)	Peak HRR (kW/m^2^)	LOI	Ignition Time (s)	Ref.
paraffin/HDPE	organophilic montmorillonite + pentaerythritol + melamine phosphate	54.58 (~55)	~10 (700)	80.06	296.7	N/A	36	[18]
paraffin/HDPE	expandable graphite + ammonium polyphosphate	93.84 (55.43)	~17 (700)	N/A	85.8	N/A	N/A	[19]
paraffin/HDPE	expanded graphite + IFR (ammonium polyphosphate, pentaerythritol, melamine)	73.6 (50.58)	~18 (350)	81	430.36	N/A	38	[20]
parafin/HDPE	iron + IFR (ammonium polyphosphate, pentaerythritol, melamine)	71.15 (~55)	N/A	83.7	274.03	N/A	30	[21]
paraffin/EPDM	nanostructured magnesium hydroxide + red phosphorus	53.57 (55.96)	~27 (700)	N/A	N/A	24	N/A	[22]
paraffin	tri-(triethoxysilylpropyl) phosphamide	74.27 (53.01)	32.4 (600)	N/A	276 W/g	N/A	N/A	[23]
the eutectic mixtures of solid and liquid paraffins with polypropylene	triazine char-forming agent + ammonium polyphosphate	126.8 (24.8)	18.8 (600)	68.3	135.9	32.8	7	[24]
paraffin	expanded graphite + ammonium phosphate	83 (20.2)	N/A	N/A	N/A	N/A	25	[25]
paraffin/HDPE	expanded graphite + magnesium hydroxide + aluminium hydroxide	35.8 (68)	26.2 (600)	103.0	655.9	N/A	38	[26]
paraffin	expanded graphite/acrylic resin	N/A	19	122	392.5	31.8	N/A	[27]
paraffin	expanded graphite + ammonium polyphosphate + carbon-forming agent + kaolinite	81.2	27.2 (600)	89.3	313.1	37.6	N/A	[28]
paraffin	expanded graphite + ammonium polyphosphate + carbon forming agent + kaolinit	121 (57.28)	22.3 (800)	N/A	N/A	31.1	N/A	[29]
palmitic acid	melamine	85.11 (62.58)	23.92 (700)	N/A	N/A	N/A	N/A	[30]
1-tetradecanol 1-hexadecanol 1-octadecanol	phosphorus and silicon—synergistic	81.5 (29.27) 107.4 (44.61) 116.9 (52.88)	18.0 15.8 16.3	N/A	N/A	N/A	N/A	[31]
capric acid + myristic acid capric acid + palmitic acid	hydromagnesite	53 (22) 56 (19)	N/A	N/A	N/A	N/A	14 9	[32]
capric acid + myristic acid capric acid + palmitic acid	magnesium hydroxide	55 (24.4) 55 (23)	N/A	N/A	N/A	N/A	24 32	
phosphorus-grafted hexadecanol	pentaerythritol phosphate	148.4	19.8 (800)	125.2	679.2	N/A	142	[33]
stearic acid	nano magnesium hydroxide + graphite powder	110.05 (85)	N/A	N/A	N/A	N/A	5	[34]
lauric acid	resorcinol bis(diphenyl phosphate) + expanded perlite	86.02	38.8 (500)	10.82	324	N/A	8	[35]
capric acid	halloysite nanotube modified by DOPO	113.52 (32.95)	24 (600)	93.95	572.65	N/A	N/A	[36]
PEG	organosiloxane (tri-(triethoxysilylpropyl) phosphamide)	124.7 (56.4)	13.5 (600)	N/A	N/A	N/A	N/A	[37]
PEG-based PU	tetrabromobisphenol-A + decabrominated-dipheny ethane	86.69 (56.3)	4.6 (650)	N/A	N/A	21.03	N/A	[38]
wood flour supported PEG	expandable graphite	32.2 (60.2)	24 (800)	99	188	30.5	N/A	[39]
PEG-based PU	dopamine-decorated black phosphorus nanosheets	127.3 (52.3)	6.14 (700)	19.2 kJ/g	442.3 W/g	24.3	N/A	[40]
PEG/epoxy resin	expanded graphite + magnesium hydroxide + zinc hydroxide	N/A	26.69 (600)	119.4	501.3	29.01	N/A	[41]
PEG-based PU	tri-maleimide end-capped cyclotriphosphazene	81.5 (51.1)	5.75 (600)	17.29 kJ/g	362.8 W/g	23.9	N/A	[42]
PEG	polyvinyl formal	145.2 (55.79)	~5 (600)	N/A	N/A	N/A	N/A	[43]
PEG	MXene/PI aerogels	167.9 (62)	3.3 (800)	21.4 kJ/g	529.3 W/g	N/A	N/A	[44]
polyrotaxane	biomass phytic acid	60.9	16.5 (600)	44	298	28.02	N/A	[45]
PEG + N,N’-Methylenebisacrylamide	microcapsule-coated ammonium polyphosphate + expanded graphite	76.34	~22 (700)	60.09	452.23	32.6	85	[46]
n-eicosane/gelatin +sodium alginate	clay nanoparticles	97.08 (35.57)	19.37 (400)	N/A	N/A	N/A	N/A	[47]
n-octadecane/PMMA	diethyl bis(2-hydroxyethyl acrylate)amino methylphosphonate (DEAMP)	109.1 (32)	13.7 (900)	204.9	501.4	25.01	N/A	[48]
n-octacosane/cellulose nanofiber	(2D)-layered black phosphorus (BP) nanosheets	251.6 (66)	5.47 (700)	37.21 kJ/g	621.2 W/g	23.9	N/A	[49]
octadecane + SiO_2_ shell	tributylphosphate	124.6 (28.1)	15 (400)	30.2 kJ/g	460.9 W/g	N/A	N/A	[50]
1-octadecane	biobased magnesium phytate	118.0 (60.1)	28.1 (700)	53.1	761	21.6	N/A	[51]

### 4.1. Modified Flame-Retarded Paraffins

One of the most commonly used phase change materials are paraffins, which are saturated hydrocarbons with the CnH_2n+2_ formula. They exhibit high latent heat storage capacities over a small temperature range and insignificant supercooling. In addition, they are non-toxic and ecologically harmless. However, the feature that needs to be improved for paraffins is their flammability [4,52,53]. Hence, Cai et al. [18] obtained a form stable phase change material based on paraffin (60%), high-density polyethylene (HDPE, 15%), organophilic montmorillonite (OMT, 10%), and intumescent fire retardant (IFR) composed of pentaerythritol (PER) and melamine phosphate (MPP). Based on the TGA results, PCM produces a large amount of char residue at 700 °C, which increases the ‘trapping’ effect of the decomposition products and, as a result, decreases the amount of flammable volatiles released. The synergistic effect of IFR and OMT was also confirmed; nanoclay reacts most likely with MPP to form an aluminophosphate and a ceramic-like structure, which results in an increase in the efficiency of intumescent char layer formation. The results of the cone calorimeter test confirmed a significant drop in the maximum heat release rate from 884.2 kW/m^2^ to 296.7 kW/m^2^ and a mass loss rate of approximately three times for the flame-retarded form stable PCM compared to the HDPE/paraffin sample. Importantly, DSC results showed that the addition of IFR and OMT did not have any significant influence on the latent heat of the stable PCM.

Later, the same research group conducted a study on flame-resistant halogen-free form-stable phase change materials [19]. Paraffin was used as the PCM, while HDPE acted as the supporting material. The researchers used an intumescent flame-retardant system with expandable graphite (EG) and ammonium polyphosphate (APP) as synergistic additives. Based on the DSC results, the fire-retardant additives did not cause any significant changes in the thermal energy storage properties, while the latent heat and the phase change temperatures of the paraffin-based composites showed better recurrences during the freezing process. Thermogravimetric analysis revealed that the addition of the halogen-free flame retardant to the form-stable PCM composite caused an increase in the amount of char residue produced up to approximately 17% at 700 °C. Cone calorimeter tests indicated that the maximum heat release rate dropped distinctly from 108.7 kW/m^2^ to 85.8 kW/m^2^. The decrease in the PHRR and the structure of the charred layer after combustion confirmed that a synergistic effect occurred between expandable graphite and ammonium polyphosphate, which led to an improvement in the flammable properties of the obtained composites. During the same study, the effect of zinc borate in combination with EG was also tested, but the results were not satisfactory. Similar research, including the addition of EG and APP to paraffin, was conducted by Sittisart and Farid [7]. The tested material showed 20% mass residue at 600 °C and a substantial reduction in peak heat release rate by about 25%.

Along this line of interest, Zhang et al. [21] obtained a fire-retardant shape-stabilized PCM based on paraffin/HDPE with an intumescent flame retardant and iron as additives. The IFR included ammonium polyphosphate, pentaerythritol, and melamine. The introduction of iron into the paraffin/HDPE/IFR (3:1:1) system improved the fire retardancy of the composite, which is the result of the synergistic effect between iron and IFR. Both the maximum heat release rate and the total heat release rate decreased due to iron addition, from 627.35 kW/m^2^ to 274.03 kW/m^2^ and from 90.4 MJ/m^2^ to 83.7 MJ/m^2^, respectively. The 3% addition of iron caused a small decrease in the latent heat of the composite from 79.91 J/g to 71.15 J/g but did not shift the temperatures of the phase change. Moreover, the thermal conductivity of the FSPCMs improved with the introduction of iron because of its high thermal conductivity.

**Figure 3:**
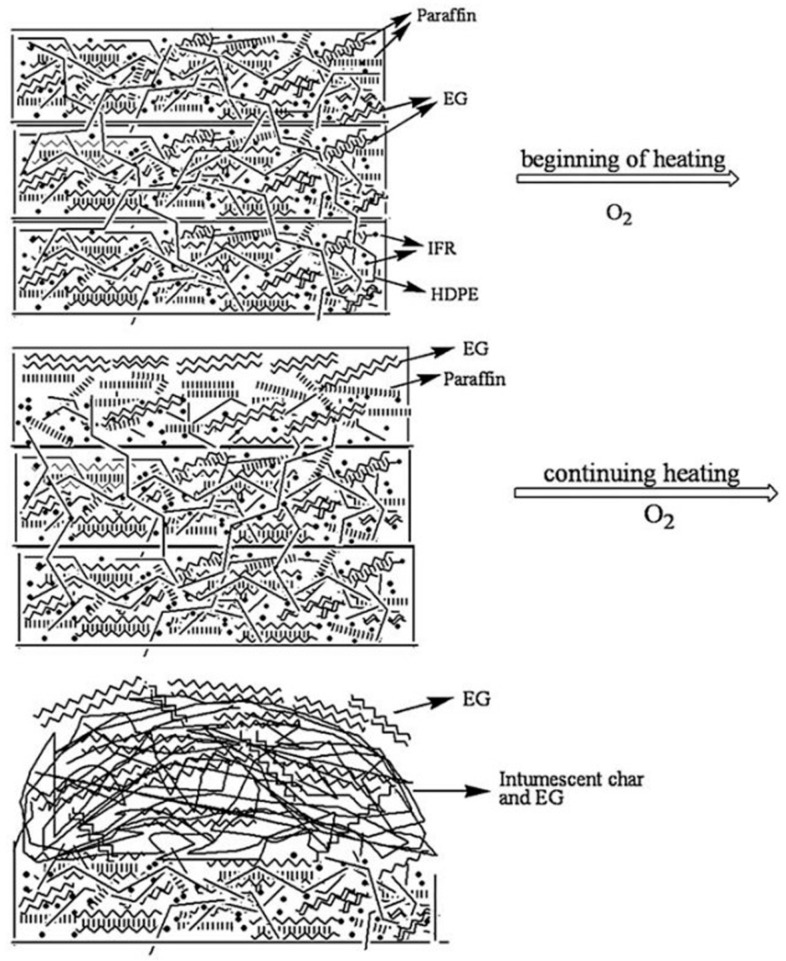
Scheme of possible combustion mechanism for paraffin/HDPE/IFR/EG as shape-stabilized PCM. Reprinted with permission from Ref. [20]. 2010, Elsevier.

In another development of this research team, a paraffin-based shape-stabilized PCM was obtained with HDPE acting as the supporting material, while expanded graphite and intumescent flame retardant (APP + PER + MA) were added to improve fire-retardant properties and thermal conductivity [20]. The thermal storage capacity of the modified PCM was lower than the theoretical value calculated by multiplying the percentage of the mass of paraffin due to the restricted molecular mobility of paraffin in the presence of additives. After the combustion process, the char residue significantly increased compared to that of the material without modifiers. The cone calorimeter test proved the enhancement of the fire retardancy properties as the peak heat release rate decreased from ca. 1130 to 430 kW/m^2^ and the total heat release rate dropped from 101 to 81 MJ/m^2^. It was postulated that at the beginning of the heating process, the first char layer could be formed by the expanded graphite; then, interactions between EG and the paraffin/HDPE/IFR intumescent char layer support formation of a more compact and dense char layer—Figure 3.

Form-stable PCMs with paraffin, EPDM as the supporting material, nanostructured magnesium hydroxide, and red phosphorus (3%) as fire retardants were obtained and investigated to ensure the synergistic flame retardancy effect of magnesium hydroxide (MH) and red phosphorus [22]. The DSC results showed that the incorporation of flame-retardant additives did not result in a significant change in the latent heat value. On the basis of the TGA results, the addition of nanostructured magnesium hydroxide to the form-stable PCMs not only significantly improved the thermal stability but also increased the flame retardancy owing to the creation of a MgO layer during combustion. Magnesium hydroxide led to the formation of char layers on the surface, which isolated the underlying material and impeded the escape of volatile decomposition products. The LOI test also confirmed that the flammability properties of the form-stable phase change materials improved as the limiting oxygen index increased substantially from 17 to 24%.

Qian’s group used an in situ sol-gel process to obtain a novel halogen-free fire-resistant phase change composites with paraffin as the thermal energy storage material and tri-(triethoxysilylpropyl) phosphamide as the sol-gel precursor [23]. The DSC results indicated that the addition of tri-(triethoxysilylpropyl) phosphamide to the paraffin caused a significant drop in the latent heat of melting from 182.3 kJ/kg to 74.27 kJ/kg as a result of the decrease in the percentage of PCM in the composite. On the basis of the TG and pyrolysis combustion flow calorimetry results, the implementation of a flame retardant in the composite improved the thermal stability and flammable properties. Hybrid silsesquioxane caused the extensive formation of an intumescent protective layer. The char residue at 600 °C was 32.4 wt% in a nitrogen atmosphere, while the peak of the heat release rate dropped by 62.1% in comparison to the pure paraffin.

The effect of incorporation of a new triazine char-forming agent and ammonium polyphosphate as intumescent fire retardants into eutectic mixtures of solid and liquid paraffins as phase-change materials with polypropylene as the supporting material was studied by Li et al. [24]. Based on the DSC results, it was concluded that for flame-retarded PCM, the latent heat increased from 116.9 kJ/kg to 126.8 kJ/kg, while the phase change temperature decreased from 30.2 °C to 24.8 °C in comparison to the nonmodified material. TG curves showed that fire retardants improved the thermal stability of the composite and significantly enhanced the char layer formation ability—the char residue raised from 0.6% to 18.8% at 600 °C. Moreover, the UL-94 test of the flame-retarded mixed paraffin/PP passed the V-0 rating, while the Limited Oxygen Index value increased from 18.8% to 32.8%. The cone calorimeter test indicated that IFR substantially decreased the maximum heat release rate from ca. 1190 to 136 kW/m2, the total heat release rate from 78.7 MJ/m^2^ to 68.3 MJ/m^2^, and also suppressed the smoke production rate.

Palacios and co-workers [32] tested different types of fire retardants (APP, hydromagnesite, magnesium hydroxide, and aluminum hydroxide) for paraffin PCMs, and found that ammonium phosphate outperforms other FRs. Consequently, in the next work, this team of authors prepared fire-retarded paraffin composites using ammonium phosphate and expanded graphite [25]. On the basis of the DSC results, the additives caused a drop in the thermal storage capacity owing to the smaller percentage of paraffin in the composition. The fire reaction behavior was characterized using a dripping test; the combustion time decreased 15 times, from 300 to 21 s. The final composite was stable even after 1000 thermal cycles. The self-extinguishing behavior was indicated by the flammability properties of the modified phase-change composite. A synergistic effect was demonstrated in the case of the combination of the phosphate flame retardant and expanded graphite, which significantly improved the flame retardancy of the PCM as well as its thermal conductivity.

Expanded graphite was also utilized by Zhou et al. [26], who prepared composite material consisting of paraffin as PCM, high-density polyethylene as the supporting material, and a flame retardants system composed of expanded graphite, magnesium hydroxide, and aluminum hydroxide. Thermogravimetric analysis indicated that PCM with the addition of MH and ATH was characterized by better thermal stability and carbonization ability as the char residue increased from 17.6% to 26.2%. The SEM microphotographs confirmed that the implementation of hydroxides caused the formation of the oxide thin layer on the surface of the mixed paraffin/HDPE, which, combined with the char layer generated through expansion of EG, created an effective physical barrier that hinders the transfer of oxygen and heat. Importantly, the presence of magnesium and aluminum hydroxides did not affect the phase change temperature or latent heat value. The cone calorimetry data proved that MH and ATH could exert a synergistic fire retardant effect with EG. The incorporated flame retardants decreased the peak heat release rate from ca. 1570 to 656 kW/m^2^, the total heat release rate from 120.8 MJ/m^2^ to 103.0 MJ/m^2^, and also suppressed the production of CO, CO_2,_ and smoke.

Another system incorporating expanded graphite was developed by Zhang and co-workers [28], who fabricated a novel fire retardant phase change material composed of paraffin, expanded graphite, ammonium polyphosphate, red phosphorus (RP), and epoxy resin (ER) for battery module. The best flame-retardant effect was obtained for the PCM composite with an APP/RP ratio of 23/10. The composite exhibited excellent heat transfer performance and relatively high latent heat value. The fire-retarded PCM passed the UL–94 V–0 test, and the LOI value increased significantly from 18.4% to 37.6%. Based on the thermogravimetric analysis data, the char residue of the composite at 600 °C rose multiple times—from 2.9% for the reference sample to 27.2% for the composite. A high-quality carbon layer with a dense and homogeneous structure yielded effective insulation of oxygen and heat during combustion. The cone calorimetry test indicated that the peak heat release rate decreased from 870.9 kW/m^2^ to 313.1 kW/m^2^ after the addition of flame retardants. Furthermore, the total heat release rate decreased by 17.8% to a value of 89.3 MJ/m^2^ value. This research showed that the combination of APP and RP results in a synergistic effect, finally leading to a reduction in the combustion rate and heat release.

**Figure 4:**
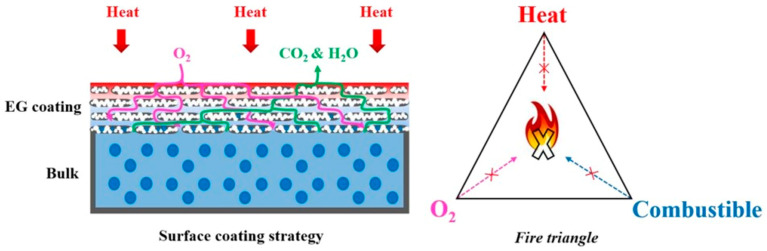
Scheme of the flame-resistant mechanism of surface-coated SSPCM. Reprinted from [27].

Xu et al. [27] conducted research on flame retardant coatings to enhance the flammability of shape-stabilized phase change materials. SSPCMs were prepared as styrene-butadiene-styrene copolymer (SBS)/HDPE/paraffin/OMMT composites. Different types of coatings were tested, but the best results were obtained for EG coating consisting of acrylic resin and expanded graphite—Figure 4. PCM-EG acquired the VO fire retardancy level, while the LOI value increased from 18.7% to 31.8%. According to the PCM-EG cone calorimeter data, the maximum heat release rate decreased from 1137 to 392.5 kW/m^2^, while the total heat release rate increased from 109.3 to 122 kJ/m^2^. Moreover, the combustion time significantly increased with lower total smoke production. The application of EG coating held back the volatilization of paraffin at an average temperature. With an increase of temperature, the coating has been expanding and the thick porous carbon layer was formed that acts as barrier for oxygen, heat, and combustibles transfer between the material and the environment.

Another filler tested in FR compositions was kaolinite—Cui et al. [29] prepared flame-retarded composite phase change material (FRPCM) containing paraffin, expanded graphite as a high thermal conductivity additive and support material, while as a flame retardant, ammonium polyphosphate, a carbon-forming agent (CFA), and kaolinite were applied. The material with given proportions of components exhibited the best properties. Thermogravimetric analysis indicated that the addition of a flame retardant significantly improved the thermal stability of the FRPCM. The residual carbon content of the PCM at 800 °C increased significantly from 2.3 to 22.3%. Furthermore, the UL-94 of the flame-retarded composite passed the V-0 rating, while the LOI value increased from 18.2% to 31.1%. Finally, it was concluded that kaolinite had a synergistic effect with an intumescent flame retardant on the flammability properties of the obtained composite material.

### 4.2. Modified Flame-Retarded Fatty Acids and Alcohols

Fatty acids, higher alcohols, and their mixtures have recently attracted increasing attention as phase change materials for various applications. This is because they are characterized by relatively high latent heat, small changes in volume, and high thermal and chemical stability after repeated cycles [54,55]. However, these organic materials are flammable. Therefore, intensive research efforts have been focused on the fire retardancy of PCMs based on these types of compounds [30,34].

Hence, Fang et al. [30] applied the sol-gel method to prepare palmitic acid/silicon dioxide (SiO_2_) thermal energy storage composite materials with melamine as a flame retardant. Palmitic acid was homogenously dispersed in the porous network of SiO_2_ due to the capillary and surface tension forces. These forces also prevent the leakage of melted acid during the phase transition. During the combustion tests, melamine released gas products, such as N_2_, NH_3,_ and H_2_O, and decomposed to yield a carbonaceous/silicate-rich char layer. Formed after the combustion process, the layer was homogeneous and dense, preventing the release of decomposed products and ultimately resulting in a decrease in the flammability of the composite. Based on DSC data, as a result of the addition of melamine, the latent heat of palmitic acid dropped from ca. 178 to 85 kJ/kg, while the temperature of the process decreases slightly by 2 degrees to 62.58 °C. The addition of melamine improved the thermal stability and fire retardancy of PCM composites.

Form-stable phase change materials based on higher alcohols (1- tetradecanol, 1-hexadecanol, and 1-octadecanol), and modified by diethyl phosphite and 2,3-epoxypropoxy propyltrimethoxysilane, were investigated in terms of flammability [31]. The incorporated additives accelerated the formation of char layers, which could isolate oxygen and heat to prevent a further burning process. Thermogravimetric analysis indicated that the thermal stability increased, and the DSC results confirmed that the modified PCMs could store and release thermal energy in the range of 29–53 °C, while the latent heat was 81.5–116.9 J/g. The synergistic flame-retardant effects of phosphorus and silicon were confirmed. As a result of their improved thermal stability and permanent fire retardancy, these materials are considered to be more competitive than other organic PCMs.

**Figure 5:**
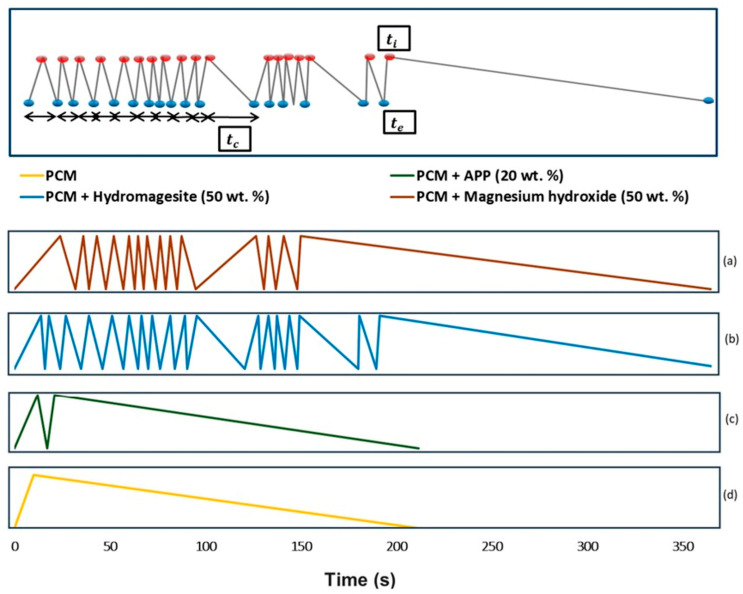
Legend of the parameters to comprehend the graph (above) and ignition-extinction periods for CA + MA mixture formulation with flame retardants (below). (**a**) Fire performance of capric and myristic eutectic with 50 wt% magnesium hydroxide; (**b**) Fire performance of capric and myristic eutectic with 50 wt% hydromagnesite; (**c**) Fire performance of capric and myristic eutectic with 20 wt% APP; (**d**) Fire performance of capric and myristic eutectic mixture. t_i_: ignition time; t_e_: extinction time; t_c_: combustion time. Reprinted from [32].

To improve the fire retardancy of PCM eutectic mixtures (~3:1) of capric acid (CA) + myristic acid (MA) and CA+ palmitic acid (PA), hydromagnesite and magnesium hydroxide (endothermic fire retardants) were used [32]. The addition of FR significantly reduced the combustion time—Figure 5. Furthermore, it is very likely that during the dripping test, the release of flammable gases occurs over a wide range of temperatures, which is why fire retardants that act at higher temperatures or in a broader range of temperatures are preferred. However, the DSC results indicated that the melting enthalpy of PCMs with the addition of fire retardants decreased almost three times, while the melting temperature did not change significantly compared to that of pure PCMs. The results indicated that the addition of 50 wt% hydromagnesite or magnesium hydroxide to the fatty acid mixtures significantly improved their fire retardancy.

The above Figure 5 shows the results of the dripping test for a eutectic mixture of capric and myristic acid. The peaks represent the time when the material ignites (t_i_) while the minimums correspond to the extinction time (t_e_). Combustion time (t_c_) is the time between ignition and extinction of the sample. A comparison of the samples (Figure 5c,d) indicates that the addition of the APP does not cause any remarkable changes in the combustion process of the material. The once-ignited PCM burns until the fuel supply comes to an end. The increased number of peaks (Figure 5a,b) is related to the numerous ignitions and extinction effects of the tested sample. These results indicate that the magnesium hydroxide (Figure 5a) and hydromagnesite (Figure 5b) are capable of extinguishing the flame of the PCM [32].

Chen et al. [33] blended a phosphorus-grafted hexadecanol (80 wt%) with pentaerythritol phosphate (PEPA, 20 wt%), an intumescent fire retardant with a cage-like structure, being both a carbon source and an acid source. The obtained FRPCM was characterized by a large heat storage capacity (186.9 J/g), good thermal stability, and high decomposition temperature (265.1 °C). The mixing of P-grafted hexadecanol with PEPA caused a synergistic effect on flame retardancy. During ignition, the grafted phosphorus groups reacted with the PEPA carbon chains, which supported the formation of char residues during combustion. SEM images confirmed the formation of a thick porous char layer. PEPA combined with phosphorus-grafted hexadecanol resulted in an extension of ignition time and decreased total heat release and maximum heat release rate by more than 50% compared to pure hexadecanol. Self-extinguishment testing showed that the flame-retardant phase change material was extinguished in a much shorter time than alcohol without modification.

Stearic-acid-based PCM, flame-retarded by graphite powder (GP) and magnesium hydroxide (nanoparticles and micro-sized), have been recently investigated by Han et al. [34]. GP (1 wt%) and MH (5 wt%) with 0.8 µm or 200 nm particle diameter were used to improve PCM fire retardancy and thermal conductivity. Both the magnesium hydroxide nanoparticles and microparticles reduced the flammability of stearic acid, whereas the effect was more profound in the case of nanoparticles. Moreover, the addition of graphite also reduced the combustion time from 41 s to 15 s and extended the time of ignition from 3 s to 5 s. The addition of MH caused a drop in the latent heat of PCM from ca. 171 to 125 kJ/kg for nano-sized, and to 89 kJ/kg for micron-sized MH while the melting temperature increased from 63 to 89 °C, and to 79 °C, respectively. Generally, the results of the burning test indicated that the introduction of nanoMH and GP reduced the flammability of PCM stearic acid by more than 60%, while the thermal conductivity of PCM increased by 20% due to the addition of GP.

Recently, Alkhazaleh’s group [35] has investigated lauric acid PCM with resorcinol bis(diphenyl phosphate) (RDP) as a flame retardant and expanded perlite (EP) as a supporting material. The optimal composition was determined as 90 wt% LA and 10 wt% RDP. EP absorbed 60 wt% of the flame-retardant phase change material. In accordance with the DSC results, the melting temperature of the composite was similar to that of the pure lauric acid. However, the heat of fusion decreased significantly, which is the result of the lower LA content in the composite. Based on the TGA results, lauric acid was thermally stable up to 120 °C, and the addition of RDP improved this property, as the flame retardant was stable up to 210 °C. The modified PCM showed advantageous thermal properties and good fire resistance. RDP significantly decreased the PHRR, THRR, and burning time of lauric acid. Furthermore, the addition of expanded perlite caused a further reduction in the flammability of the composites tested.

In a very recent development, Cheng et al. [36] synthesized microencapsulated phase change material (mPCM, capric acid/PMMA) with incorporated halloysite nanotubes (D-HNT) previously modified by 9,10-dihydro-9-oxa-10-phosphaphenanthrene-10-oxide (DOPO). Composites with D-HNTs showed better thermal stability, and for those samples, a higher residual mass of 24% at 600 °C was also observed in comparison to 5.5% for pure mPCM. Latent heat during the melting decreased from ca. 124.8 J/g (mPCM) to 113.6 J/G (D-H-mPCM) while the phase transition temperature remained at nearly the same level. Based on the cone calorimeter test, it was concluded that the D-H-mPCM showed the smallest peak of heat release rate and total heat release of 572.65 kW/m^2^ and 93.95 MJ/m^2^, respectively. These results suggest that the addition of halloysite nanotubes modified by DOPO effectively enhanced the fire retardancy of the mPCM. Furthermore, the research showed that the incorporation of D-HNTs into mPCM improved the thermal conductivity of the obtained composites.

### 4.3. Modified Flame-Retarded Polymers

Polymeric PCMs play an important role in low-temperature thermal energy storage. In the case of polymers, the key parameter that determines the temperature and heat of fusion is its crystallinity. The best candidates for PCM from the group of macromolecular compounds are polymers with straight, unbranched chains capable of crystallization, such as some polyolefins and polyethers. They can be easily tailored to the desired phase temperature range by selecting polymers with defined average molecular weight and melting/crystallization temperatures. Among various polymers, special attention was paid to poly(ethylene glycol) (PEG) [56,57,58]. However, it belongs to combustible materials, and there is a need to flame-retard PEG-based PCM systems.

**Figure 6:**
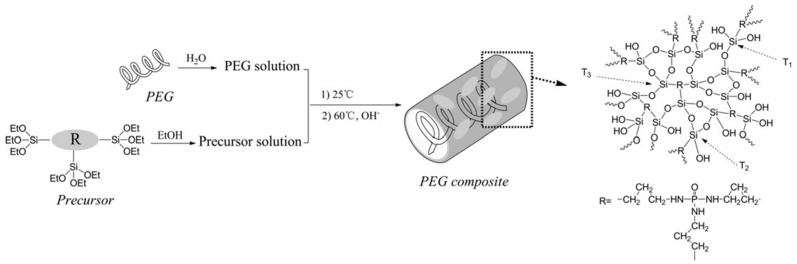
Illustration of the preparation process of the PEG/silsesquioxane composite. Reprinted with permission from Ref. [37]. 2013, Elsevier.

Hence, Qian et al. [37] studied PEG/silsesquioxane composites obtained by using the sol–gel process—Figure 6.

The authors found that PEG/silsesquioxane composites were characterized by a large latent heat of the phase transition of ca. 125 kJ/kg, and good thermal reliability after thermal cycling for 1000 cycles, as well as an increased thermal stability. A distinct flame retardancy effect was observed—the peak heat release rate (PHRR) of PEG/silsesquioxane system decreased by 38.6% while time to PHRR increased by 31 s compared with unmodified PEG.

Guo et al. [39] studied the shape-stabilized composite PCM based on PEG that was obtained by vacuum impregnation of wood flour (WF) with melted PEG 4000 (WF-s-PEG). Next, WF-s-PEG was mixed with polypropylene (PP) to prepare wood-plastic PCMs. Additionally, expandable graphite was incorporated to obtain flame-retarded PCM (FR-PCM). It has been found that FR-PCM showed higher heat (164 J/g) and phase change temperature (60 °C). The incorporation of EG allowed for improving the thermal conductivity and flame retardancy of the PCM composite.

**Figure 7:**
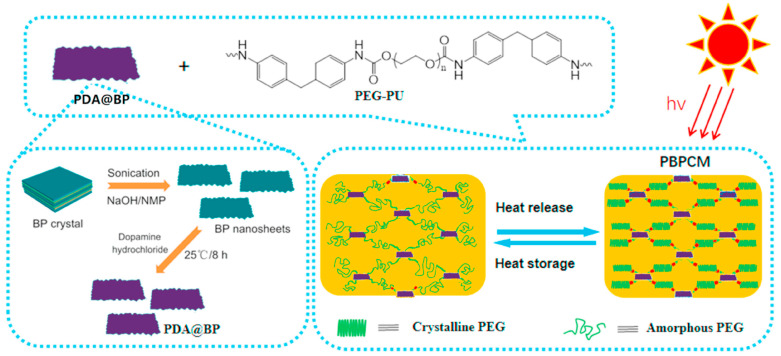
Schematic illustration of PBPCMs. Reprinted with permission from Ref. [40]. 2021, Elsevier.

Poly(ethylene glycol)-based polyurethane (PEG-PU) form stable PCMs modified with dopamine-decorated black phosphorus nanosheets (PDA-BP) were studied by Du et al. [40]—Figure 7.

PDA-BP act as photothermal fillers that allow solar-thermal energy conversion, while BP nanosheets can form a protective carbonaceous layer on the combusted surface and enhance the flame retardancy of the obtained PCM system. The experiments performed revealed that introducing PDA-BP into PEG-PU improved the solar thermal conversion efficiency (up to 88.5%), as well as the thermal conductivity. Additionally, a significant decrease in total heat release and heat release rate was found by microscale combustion calorimetry.

Chen et al. [43] proposed PCM based on the poly(ethylene glycol) shape stabilized with poly(vinyl formal) (PVF). The phase transition enthalpy of the PEG/PVF systems ranges from 104.1 to 158.4 J/g. Moreover, an increase in the char residue and thermal stability was observed as compared to unmodified PEG.

**Figure 8:**
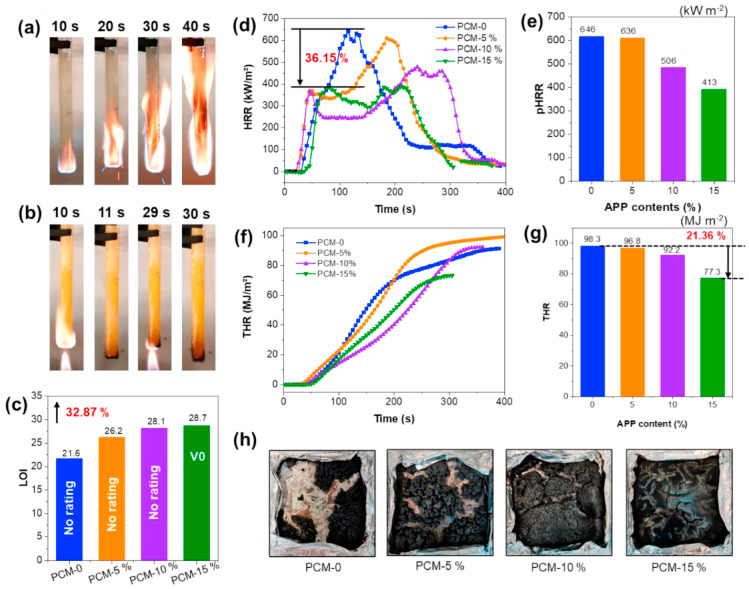
(**a**) Digital photos of UL-94 test for sample PCM-0, (**b**) Digital photos of UL-94 test for sample PCM-15%, (**c**) LOI values of the samples (the LOI value increased by 32.87% for PCM-15%), (**d**) heat release rate (HRR) curves, (**e**) peak of HRR (pHRR) values, (**f**) THR curves, (**g**) THR values based on the THR curves, and (**h**) char images of the four samples after CONE tests. Reprinted with permission from Ref. [59]. 2022, Elsevier.

PEG modification with ammonium polyphosphate (APP) and poly(glycerol-itaconic acid) was performed by Yin et al. [59], who found that with an increase in APP load, limiting oxygen index (LOI) values significantly increased from 21.6% to 28.7% after incorporation of 15 wt% APP (Figure 8). In vertical tests the obtained material reached UL-94 V0 rating and the pHRR also significantly decreased. Moreover, the obtained PCM system exhibited form stability with the heat of phase transition higher than 70 J/g.

In another work, Cao et al. [44] proposed new leakage-proof composite PCM characterized by solar thermal conversion ability and proper flame retardancy. The authors used MXene nanosheets obtained by etching the MAX phase with lithium fluoride and hydrochloric acid solutions. Next, MXene/polyimide (PI) aerogels were prepared via freeze-drying method and then through the thermal imidization in the presence of poly(amic acid). At the next stage, the MXene/PI aerogels were infiltrated with PEG by vacuum impregnation. Shape-stable MXene/PI@PEG phase change composites (MPPCCs) with high PEG loading (up to 98.1%) were characterized by the high heat of phase transition up to 167.9 J/g and significantly improved flame retardancy.

**Figure 9:**
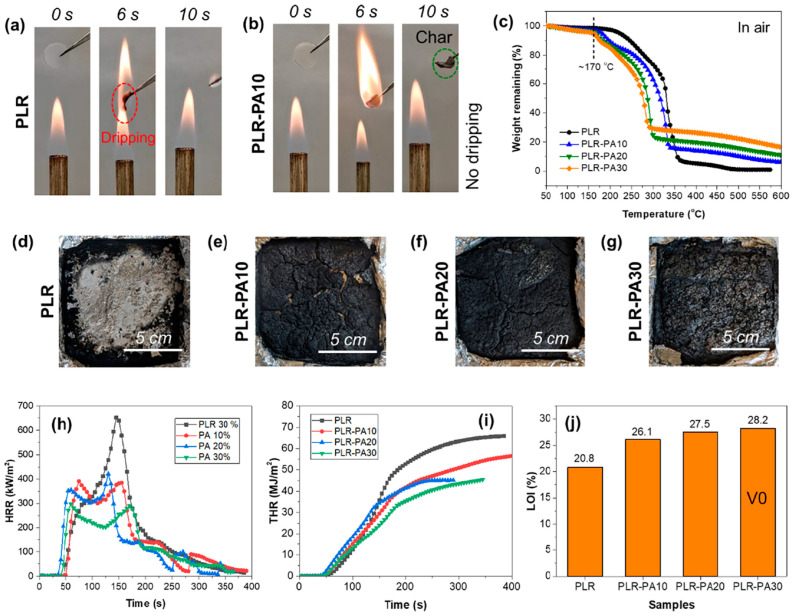
Combustion images of sample (**a**) PLR, (**b**) sample PLR-PA10, (**c**) TGA curves of all the PCM samples, (**d**) char morphology of sample PLR, (**e**) char morphology of sample PLR-PA10, (**f**) char morphology of sample PLR-PA20, (**g**) char morphology of sample PLR-PA30, (**h**) HRR curves of all the PCMs, (**i**) THR curves of all the PCMs, and (**j**) LOI values of the PCMs. Reprinted with permission from Ref. [45]. 2022, Elsevier.

Yin et al. [45] used phytic acid (PA) derived from biomass as a flame retardant for PCM based on polyrotaxane (PLR). The obtained results show that the fire safety of PLR was significantly improved after modification with PA; it was possible to achieve the pHRR decrease of 54.2%, THR decrease of 34.0%, while LOI increased from 20.8% to 28.2% as it is shown in Figure 9. Moreover, the obtained PCMs exhibited form stability without leakage, with improved thermal conductivity.

## 5. Applications of Flame-Retarded PCMs

**Figure 10:**
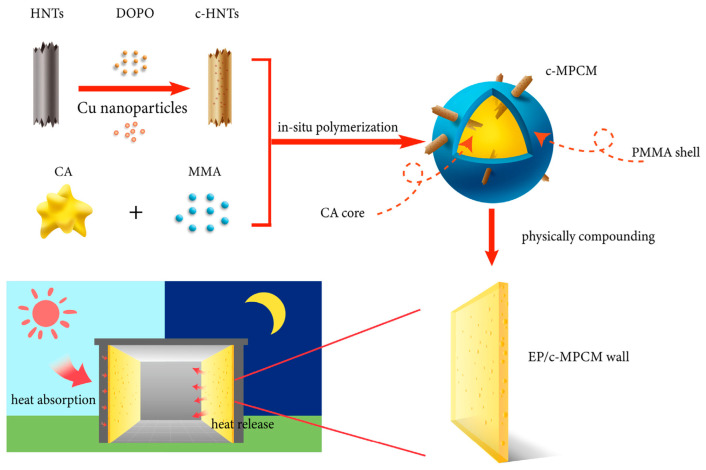
Experimental flowchart of the experiment. Reprinted with permission from Ref. [60]. 2023, Elsevier.

Flame-retarded PCMs are now mostly applied in buildings to meet fire safety standards that are crucial for people’s safety. PCMs can be incorporated into buildings’ thermal management systems both in bulk and in microencapsulated form. Organic PCMs are one of the most important groups of PCMs for buildings due to the proper temperature of phase change. However, both the organic core and organic shell of PCMs are usually flammable and the risk of combustion should be addressed, mainly by the modification of PCMs or shell materials with flame retardants. Hence, Kang et al. [60] developed microencapsulated PCMs (MPCMs) modified with halloysite nanotubes (c-HNTs) doped by 9,10-dihydro-9-oxa-10-phosphaphenanthrene-10-oxide (DOPO) and Cu nanoparticles to improve the flame resistance of PCMs microcapsules—Figure 10.

The obtained results revealed that MPCMs have better thermal stability, while DSC results indicated that they delay the heat transfer to the PCM core. MPCMs were inserted into the epoxy resin (EP) and the cone calorimetry tests show that the heat and smoke release rate of EP composite was reduced as compared to EP.

Haurie et al. [61] investigated shape-stabilized nano-enhanced phase change material (SS-NEPCM) based on EPDM as a polymeric matrix, PA as PCM, and MMT. The SS-NEPCM was prepared using a Banbury mixer. Authors observed that modification with MMT leads to an increase in thermal stability due to the barrier effect and fire resistance improvements evidenced by a substantial reduction of dripping.

**Figure 11:**
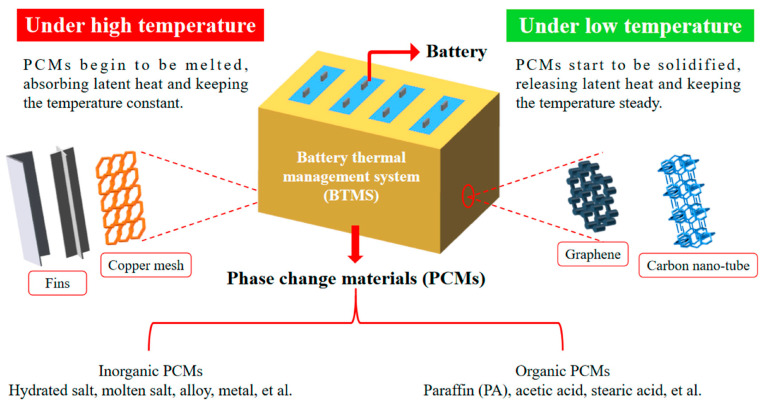
Operating principle of PCMs with thermal conductivity improvement methods such as adding fins and fillers application in BTMS under various temperatures. Reprinted from [62].

Another area of flame-retarded PCMs is to provide proper battery thermal management, especially today, as electric vehicles (EVs) play a key role in reducing carbon emissions, and their number rapidly grows. In electric vehicles, the battery pack with power lithium-ion batteries is the power source, whose performance strongly depends on the temperature and efficient battery thermal management system (BTMS). Temperature control in such systems is of primary importance, and PCM-based cooling systems are considered a promising solution that can secure a uniform temperature distribution [41,46,62,63]. However, depending on the application area of BTMS it should be systematically tested. For such purposes, it is necessary to estimate the heat release of the battery pack to obtain the corresponding heat consumption by PCM. Some of the working principles of PCMs, and different thermal conductivity improvement strategies, such as the incorporation of fins or conductive (nano)fillers to BTMS, were summarized in work by Liu et al. [62]—Figure 11.

Recently, Su et al. [64] proposed the application of flame-retarded PCM in firefighting protective clothing to provide thermal comfort and protection to firemen. Authors coated fabrics commonly used in the firefighter protective clothing fabrics with paraffin-based PCM modified with silicone and phosphorus-nitrogen (PN) flame retardants, by the dry coating method. They found that fabrics coated with PN flame-retarded PCM provided higher fire resistance, compared to the textile coated with PCM flame retarded using Si-FR. Moreover, it was observed that PCM incorporation significantly improved the thermal protection of firefighting protective clothing. Demirbağ and Aksoy [47] studied the thermal stability and flame-retardant properties of cotton fabrics modified with n-eicosane as PCM microencapsulated with clay nano-particles (Clay-NPs) doped gelatin/sodium alginate shells. The DSC results revealed that the heat of phase transition of microcapsules was in the range of 97–114 J/g, and the particle sizes of 1.37–1.60 µm. The obtained microcapsules indicated better thermal stability. Moreover, after incorporation into cotton fabrics they improved flame retardant properties of the tested fabrics.

## 6. Conclusions and Future Outlooks

Phase change materials obtain increasing attention as one of the key groups of the materials used for the thermal energy storage. They have found numerous applications in different sectors; however, organic PCMs, such as paraffins, fatty acids, fatty alcohols, and polymers, are flammable materials. This feature limits some applications, e.g., in the perspective sectors of energy efficient buildings and electric vehicles. Hence, numerous research efforts focused on the flame retardancy of PCMs by using halogenated alkanes, intumescent flame retardants, heat absorbers, and synergists. Halogenated flame retardants cause ozone layer depletion and are being gradually phased out, whereby heat absorbers, mostly magnesium hydroxide and aluminum hydroxide, need to be applied at high loads which may deteriorate the PCM’s heat accumulation efficiency. In this context, intumescent flame retardants and synergists are widely applied, and new formulations have been proposed in recent years. On the basis of new knowledge provided in the field of nanotechnology and nanomaterials, novel nanostructured additives are tested to enhance flame retardancy effects, such as graphene, nanoclays, and silsesquioxanes. They still need to be further developed by, e.g., functionalization to yield additional functions to the core material and/or surface coating. Another route of future efforts will most likely focus on a well-thought chemical modification of PCMs, including bio-based phase change materials, to avoid problems with the physical mixing of components and their limited miscibility leading to phase separation and other undesired effects. It is believed that sustainable biobased FRs which reduce environmental footprints and are dedicated to PCMs will also be developed in the future.

Future developments will probably concentrate on linking materials science and engineering approaches with chemical modifications/functionalization of flame retardants, especially nano-FRs. New fabrication routes, e.g., utilizing 3D printing technologies, will give the possibility to incorporate different FRs to the top/bottom layers to reach the optimal performance of materials with complex geometries. Such integrated and sustainable strategy shall lead to a steady increase in the use of PCMs in new sectors and to the development of novel advanced products with enhanced properties.

## Figures and Tables

**Table 1:** Different types of fire retardants and their modes of action. Reprinted/adapted with permission from Ref. [9]. 2022, Elsevier.

	Halogenated Alkanes	IFRs	Heat Absorbers	Synergists
**Examples**	Halon 1211, Halon 1301, Halon 1202, etc.	Melamine, APP, PER	Mg(OH)_2_, Al(OH)_3_	Metallic nano-particles; Nano clays
**Mechanism**	Removing free radicals from flame	Forming protective char layer; Release of non-flammable gases	Endothermic decomposition; Release of non-flammable gases	Improving performances of other fire retardants
**Advantages**	Most effective flame retardants	Medium efficacy	Low cost	Potentially high efficacy
**Drawbacks**	Environmentally unfriendly	Works best with solid surfaces rather than liquid surfaces	Lost efficacy	Requires optimization and research

**Table 2:** Common halogenated FRs, chemical structure, and additional information. Reprinted/adapted with permission from Ref. [13]. 2021, Wiley.

Halogenated FR	Code	Chemical Formula	Additional Info
Tetrabromobisphenol A	TBBPA	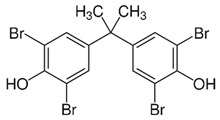	Most common halogenated FR, reactive FR in epoxy resins
Polybromodiphenylether	PBDE	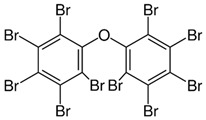	Contains 10 bromine atoms, FR additives in styrene polymers, polyolefins, polyesters, and nylons
Hexabromocyclododecane	HBCD	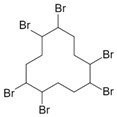	Cycloaliphatic halogenated FR, expanded, or compact PS and textiles
Tetrabromophthalic anhydride	TBPA	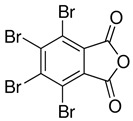	FR additive in unsaturated polyesters, base material for other FRs

## Data Availability

Not applicable.

## Abbreviations

APP	ammonium polyphosphate
ATH	aluminum hydroxide
BP	black phosphorus
CA	capric acid
CFA	carbon-forming agent
DEAMP	diethyl bis(2-hydroxyethyl acrylate)amino methylphosphonate
DSC	differential scanning calorimetry
DOPO	9,10-dihydro-9-oxa-10-phosphaphenanthrene-10-oxide
EG	expandable/expanded graphite
EP	expanded perlite
EPDM	ethylene propylene diene terpolymer
ER	epoxy resin
EVs	electric vehicles
FRs	flame retardants
FRPCM	flame-retarded composite phase change material
FSPCMs	flame retardant shape-stabilized phase change materials
GP	graphite powder
HDPE	high-density polyethylene
HFRs	halogenated flame retardants
HNT	halloysite nanotubes
IFR	intumescent flame retardants
LA	lauric acid
LOI	limiting oxygen index
MA	myristic acid
MH	magnesium hydroxide
MPP	melamine polyphosphate
OMMT	organophilic montmorillonite
PA	palmitic acid/phytic acid
PCMs	phase change materials
PEPA	pentaerythritol phosphate
PER	pentaerythritol
PET	poly (ethylene terephthalate)
PHRR	peak heat release rate
PI	polyimide
PLR	polyrotaxane
PMMA	poly(methyl methacrylate)
POSS	polyhedral oligomeric silsesquioxanes
PP	polypropylene
PU	polyurethane
PVF	poly(vinyl formal)
RDP	resorcinol bis(diphenyl phosphate)
RP	red phosphorus
SBS	styrene-butadiene-styrene copolymer
SEM	scanning electron microscopy
SSPCMs	shape stabilized phase change materials
TES	thermal energy storage
TGA	thermogravimetric analysis
THRR	total heat release rate
WF	wood flour
